# Identification of Maize Long Non-Coding RNAs Responsive to Drought Stress

**DOI:** 10.1371/journal.pone.0098958

**Published:** 2014-06-03

**Authors:** Wei Zhang, Zhaoxue Han, Qingli Guo, Yu Liu, Yuxian Zheng, Fangli Wu, Weibo Jin

**Affiliations:** 1 College of Life Sciences, Northwest A&F University, Yangling, China; 2 Institute of Bioengineering, College of Life Sciences, Zhejiang Sci-Tech University, Hangzhou, China; CSIR Institute of Genomics and Integrative Biology, India

## Abstract

Long non-coding RNAs (lncRNAs) represent a class of riboregulators that either directly act in long form or are processed to shorter miRNAs and siRNAs. Emerging evidence shows that lncRNAs participate in stress responsive regulation. In this study, to identify the putative maize lncRNAs responsive to drought stress, 8449 drought responsive transcripts were first uploaded to the Coding Potential Calculator website for classification as protein coding or non-coding RNAs, and 1724 RNAs were identified as potential non-coding RNAs. A Perl script was written to screen these 1724 ncRNAs and 664 transcripts were ultimately identified as drought-responsive lncRNAs. Of these 664 transcripts, 126 drought-responsive lncRNAs were highly similar to known maize lncRNAs; the remaining 538 transcripts were considered as novel lncRNAs. Among the 664 lncRNAs identified as drought responsive, 567 were upregulated and 97 were downregulated in drought-stressed leaves of maize. 8 lncRNAs were identified as miRNA precursor lncRNAs, 62 were classified as both shRNA and siRNA precursors, and 279 were classified as siRNA precursors. The remaining 315 lncRNAs were classified as other lncRNAs that are likely to function as longer molecules. Among these 315 lncRNAs, 10 are identified as antisense lncRNAs and 7 could pair with 17 CDS sequences with near-perfect matches. Finally, RT-qPCR results confirmed that all selected lncRNAs could respond to drought stress. These findings extend the current view on lncRNAs as ubiquitous regulators under stress conditions.

## Introduction

Maize (*Zea mays* L.) is a major cereal crop worldwide, serving as a major staple for both human consumption and animal feed. It has also become a key resource for industrial applications and bioenergy production. Drought is one of the major abiotic stresses that limit maize productivity [1]. The most effective way to stabilize and improve maize production under drought conditions is to improve the varieties in terms of drought tolerance. Significant differentiation of drought tolerance among maize genotypes implicates the hope of genetic enhancement for drought tolerance to improve maize [2]–[4]. However, breeding for drought tolerance is particularly challenging because of the genetic complexity of this trait. Drought tolerance has been well-documented to result from cooperative interactions among multiple morphological, physiological, and biochemical characters. Different genotypes may have different responses to drought stress [3], [5]–[7]. Therefore, efficient improvement requires an in-depth understanding of the gene expression regulation mechanisms in response to drought stress.

Recent genome-wide transcriptome analysis methods, such as tiling arrays and next generation sequencing, have revealed a large number of stress-responsive ncRNAs. Emerging evidence has revealed that ncRNAs are the major products of plant transcriptomes with significant regulatory importance [8], [9]. ncRNAs are transcribed from intergenic regions, antisense strands of protein-coding genes, and pseudogenes. According to their size, ncRNAs are classified as small ncRNAs (sRNAs) (<40 nt) and long ncRNAs (lncRNAs) (>200 nt). These ncRNAs are involved in the transcriptional and posttranscriptional regulation of gene expression as well as the modulation of RNA stability and translation under stress conditions [10]–[14].

In contrast to sRNAs, much less is known about the large and diverse population of lncRNAs. This heterogeneous class of transcripts generally does not contain any long open reading frame (ORF) (no ORF >70 AA). lncRNAs can interact with proteins to regulate transcription, translation, or mRNA stability [15]–[18]. Furthermore, several of these long ncRNAs are precursors of miRNAs and siRNAs [19], [20]. Similar to some miRNAs, certain lncRNAs are induced in various developmental processes as well as during abiotic stress responses in plants and animals [21]–[24]. In *Caenorhabditis elegans*, 25 lncRNAs are regulated in seven developmental stages and two stimulated conditions [25]. In *Arabidopsis*, 15 plant lncRNAs display diverse tissue-specific expression patterns and/or regulation by environmental stimuli [26]. In maize, numerous potential lncRNAs have been identified using the full-length cDNA sequences of maize, and these lncRNAs may function to regulate expression of other genes through multiple RNA-mediated mechanisms [27]. However, reports on lncRNAs involved in drought-responsive regulation in maize are lacking.

In this study, we performed genome-wide screening of drought stress-responsive maize transcripts to isolate a collection of lncRNA genes expressed in maize leaves. A total of 664 putative transcripts were identified as drought-responsive lncRNAs. Of these 664 transcripts, 8 were found to be homologous to existing maize miRNA precursors. The remaining 656 lncRNAs were classified as either small RNA precursors or other ncRNAs through alignment with other small RNA databases. In total, 62 lncRNAs were classified as both shRNA and siRNA precursors and 341 lncRNAs were classified as siRNA precursors, including 62 shRNAs. The remaining 315 lncRNAs were classified as other lncRNAs that are likely to function as longer molecules. Among these 315 lncRNAs, 10 were identified as antisense lncRNAs and 7 could pair with 17 CDS with near-perfect matches. qRT-PCR results confirmed that all selected lncRNAs could respond to drought stress. These findings extend the current view on lncRNAs as ubiquitous regulators under stress conditions.

## Materials and Methods

### Data sets

Maize genomic sequences (B73_RefGen_v2), genome annotation-filtered site file (ZmB73_5b_FGS), and the sequences of 63540 CDSs were downloaded from the ftp site: ftp://ftp.maizesequence.org/pub/maize/release-5b/. Small RNA sequences were downloaded as FASTA files from NCBI under the series GSE15286 (http://www.ncbi.nlm.nih.gov/geo/query/acc.cgi?acc=GSE15286) [28]. Files for both roots and shoots for each group (known_miRNA, shRNA, and siRNA) were combined into a single FASTA file. All of the miRNA precursor sequences were downloaded from miRBase (Version 20) [29] at the website: http://www.mirbase.org/. BLAST+ executables, version 2.2.23, was downloaded from the NCBI website for local use.

### Bioinformatics analysis

**Figure 1:**
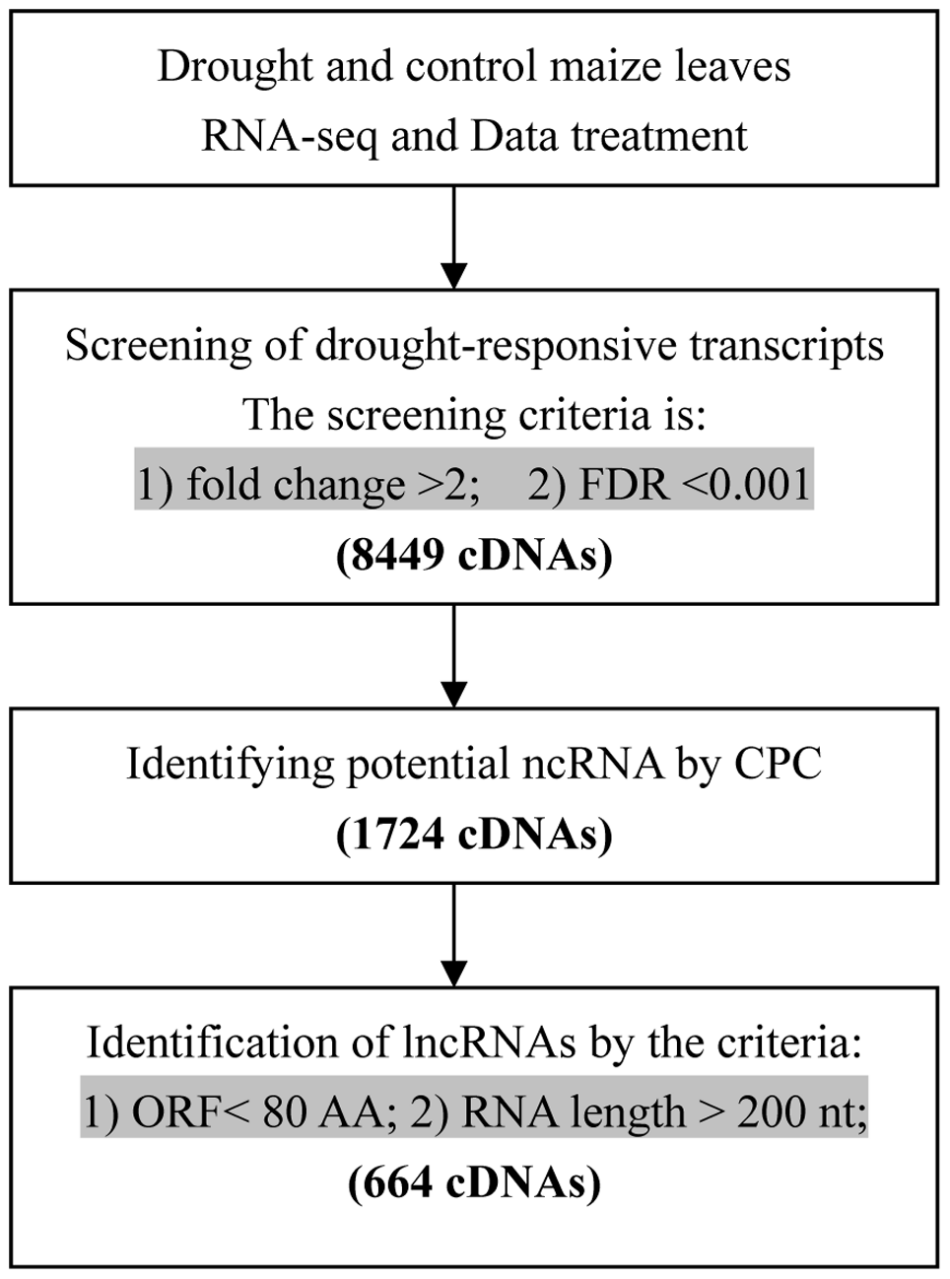
The flowchart for predicting non-coding RNA responsive to drought stress.

All sequenced reads from each sample were aligned to the maize reference genome (B73_RefGen_v2) using Tophat [30]. The transcriptome of each sample was assembled separately using Cufflinks [31]. Then the assembled transcript isoforms were compared with the maize genome annotation-filtered site (FGS), and producted a non-redundant genome features annotation file (Generic Feature Format, gff) [32]. Only reads uniquely mapped to the non-redundant gff annotated site were kept for expression analysis. The expression level of every isoform was calculated by using RPKM method (Reads per kilobase transcriptome per million mapped reads) [33]. The sequences of drought stress responsive transcripts, which were 2-fold up- or downregulated with False Discovery Rates (FDRs) [34] less than 0.001, were retrieved using a Perl script, and then were uploaded to the Coding Potential Calculator (CPC) website for classification as protein-coding or ncRNA candidates. Finally, a Perl script was written to screen these ncRNAs for RNA length >200 nt and ORF <80 AA, and drought-responsive lncRNA sequences were extracted. The identification flowchart of lncRNAs is shown in Figure 1.

To identify the drought-responsive lncRNAs as precursors of known miRNAs, the miRNA precursors were aligned to lncRNAs using BLAST. The lncRNAs matched the miRNA precursors with >99% identification accuracy; those lncRNAs with >90% coverage were finally classified as miRNA precursors.

To identify drought-responsive lncRNAs as precursors of shRNAs or siRNAs, the shRNA and siRNA libraries were respectively mapped to the lncRNAs using Bowtie without mismatches [35]. lncRNAs homologous to the shRNA reads were classified as shRNA precursors while those homologous to the siRNA reads were classified as siRNA precursors.

### Plant growth and drought stress treatment

Maize (*Zea mays* cv B73) seeds were soaked in deionized water for 12 h and then placed on a sheet of moist filter paper in a Petri dish. These seeds were germinated at 28°C for 3 d. Germinated seeds were transferred to a floating foam sheet in the hydroponic boxes (40×20×12cm^3^) containing continuously aerated water in a growth chamber (28°C day/26°C night, 16 h photoperiod, 30%–50% relative humidity) for about 1 wk. The seedlings were then cultivated in 1/2 Hoagland solution [2.5 mM KNO_3_, 2.5 mM Ca(NO_3_)_2_·4 H_2_O, 1 mM MgSO_4_·7 H_2_O, 0.5 mM KH_2_PO_4_, 50 µM Fe-EDTA, 7.5 µM H_3_BO_3_, 2.5 µM (NH_4_)_6_Mo_7_O_24_, 1.25 µM MnCl_2_, 1 µM ZnSO_4_, and 0.5 µM CuSO_4_, pH 6.0] for about another week. To induce expression of target genes, seedlings at the three-leaf stage were subjected to drought stress treatment. Drought treatment was carried out by submerging the roots of the plants in 1/2 Hoagland solution with 16% (w/v) polyethylene glycol (MW 8000) for different periods. The shoot and root tissues of the control and stressed seedlings were harvested at 3 time points (0 h, 5 h, and 10 h) after treatment in three biological replicates. These samples were immediately frozen in liquid nitrogen and stored at −70°C for studies on dynamic expression changes in the transcripts.

### RNA isolation and quantitative real-time PCR analysis

Total RNAs were extracted from leaf tissues using a TRIzol reagent (Invitrogen, Carlsbad, CA, USA), followed by RNase-free DNase treatment (Takara, Dalian, China). The RNA concentrations were quantified by a NanoDrop ND-1000 spectrophotometer.

The expression profiles of drought-responsive lncRNAs were assayed by Reverse Transcription (RT) quantitative PCR (qPCR). 500 ng of total RNAs were used for initiating the RT and the RT product was used as template for qPCR using the lncRNAs specific primers. The RT reactions were performed using M-MLV Reverse Transcriptase (Takara, Japan) according to the supplier's protocol. Primers were then added to perform PCR. The *GAPDH* was used as the internal control for RT-qPCR. All the oligos used in this study were listed in Table S1.

SYBR Green PCR was performed as per the manufacturer's instructions (Takara, Japan). Briefly, 2 µl of cDNA template was added to 12.5 µl of 2× SYBR Green PCR master mix (Takara), 1 µM concentration of each primer, and ddH_2_O to a final volume of 25 µl. The reactions were amplified for 10 s at 95°C, followed by 40 cycles of 95°C for 10 s and 60°C for 30 s. All reactions were performed in triplicate, and the controls (no template and no RT) were included for each gene. The relative expression levels were calculated using the formula: Ratio = (*E*_target_) ^ΔCp^_target_^(control-treatment)^/(*E*_ref_) ^ΔCp^_ref_^(control-treatment)^ [36].

## Results

### Identification and expression characterization of drought stress-responsive long ncRNAs in maize

A total of 8449 drought stress responsive transcripts that were 2-fold up- or downregulated with FDRs less than 0.001 were obtained from RNA-seq data (Unpublished data from Zhaoxue Han's lab). In this study, to identify putative maize lncRNAs responsive to drought stress, the 8449 RNA sequences were first uploaded to the CPC website for classification as protein coding or non-coding RNAs; 1724 RNAs were identified as potential non-coding RNAs (Data S1). A Perl script was then written to screen these 1724 ncRNAs with the RNA length >200 nt and ORF <80 AA; a total of 664 transcripts were finally identified as drought-responsive lncRNAs including 205 intragenic lncRNAs, 421 intergenic lncRNAs, 23 antisense and 15 lncRNAs overlapping with parts of inter- and intragenic sequences (Figure 1, Data S2, GenBank accession number: KJ682450-KJ682628, KJ731849- KJ732333). The expression profiles of these 664 lncRNAs obtained between control and drought stress samples were extracted from RNA-seq data (Table S2). Among the 664 lncRNAs, 567 lncRNAs containing 344 of intergenic, 189 of intragenic, 21 of antisense and 13 of overlapping lncRNAs were upregulated in drought-stress leaves. The remaining 97 were downregulated including 77 of intergenic, 16 of intragenic, 2 antisense and 2 overlapping lncRNAs (Table 1, Table S2). Moreover, 126 of the 664 drought-responsive lncRNAs showed high homology (>94% identity and >80% coverage) to known lncRNAs identified by Boerner et al. [27] and were thus considered as known lncRNAs (Table S3).

**Table 1:** Identification and expression analysis of lncRNAs responding to drought stress.

	Up-regulation	Down-regulation	total
Intergenic lncRNAS	344	77	421
Intragenic lncRNAs	189	16	205
Antisense lncRNAs	21	2	23
Overlapping lncRNAs	13	2	15
Total	567	97	664

### ncRNA transcripts correspond to miRNA precursors

**Figure 2:**
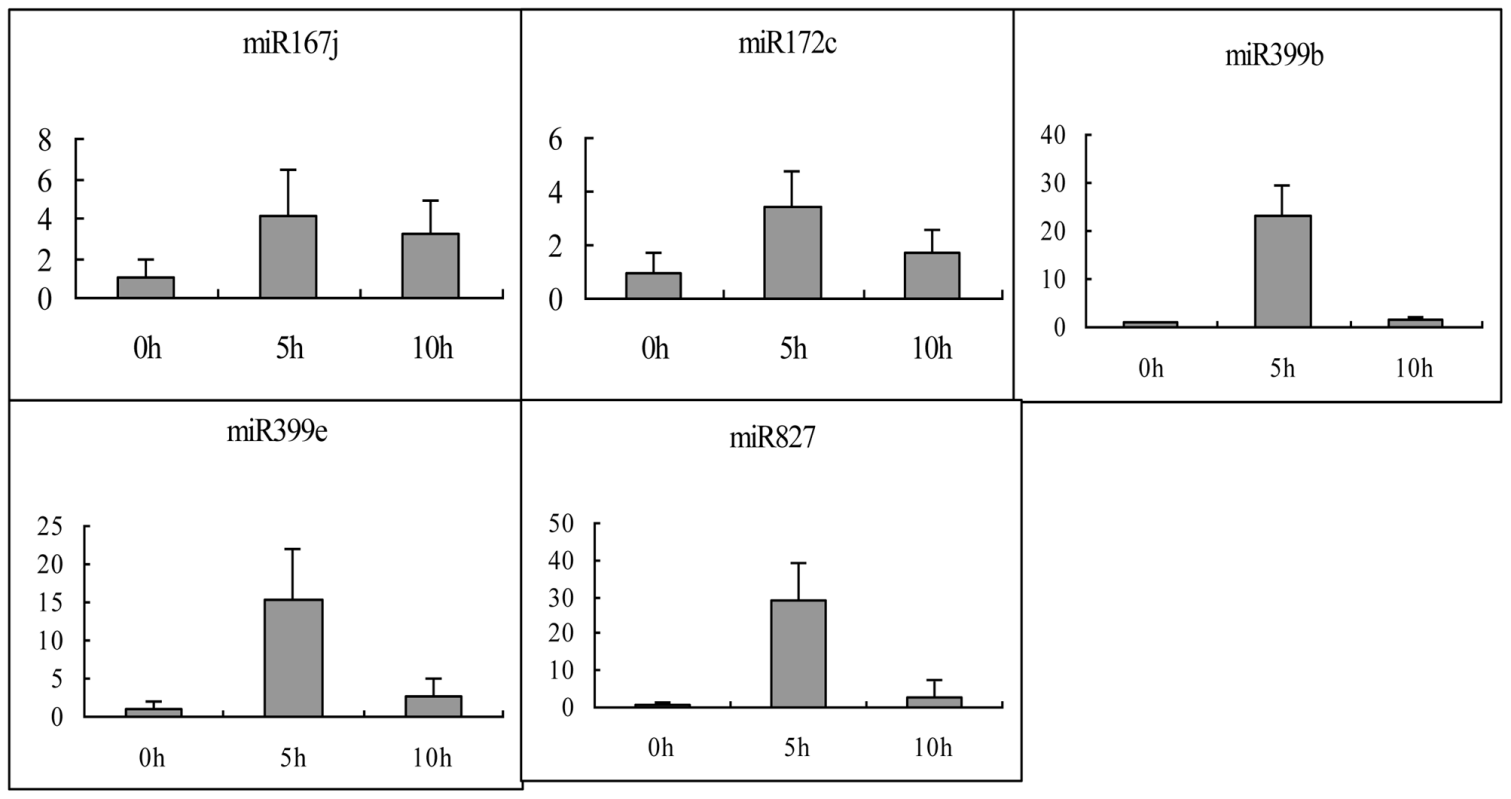
Quantitative analysis of the precursor levels of 5 miRNAs in the leaves using RT-qPCR at 0, 5 and 10 h. GAPDH RNA was used as the internal control. Error bars indicate SD obtained from three biological repeats.

By aligning miRNA precursors to the 664 long ncRNAs, we identified 8 lncRNAs as 7 known maize miRNA precursors of miR167j, miR169d, miR169h, miR172c, miR399b, miR399e and miR827 (Table 2). Among the 8 lncRNAs, GRMZM2G420571_T01 was an intragenic lncRNA and the TCONS_00054544 was an overlapping lncRNAs, and the remaining 6 were intergenic lncRNAs. To prove that these miRNA precursor lncRNAs could respond to drought stress, quantitative RT-PCT (qRT-PCR) was employed to detect their expression in both drought-stressed and control samples. The precursor sequences of miR169d and miR169h are high similarity and the expressions of them were opposite on RNA-seq data. Therefore, the expression detections for the other 5 miRNA precursor lncRNAs were performed and the results show the obviously upregulated in stressed leaves compared with control leaves (Figure 2). Moreover, only 2 lncRNAs, namely the precursors of miR167j and miR172c, exhibited consistent expression changes compared with the RNA-seq data. The remaining 3 miRNA precursors exhibited upregulation in drought-stressed leaves as determined by qRT-PCR but showed downregulation in comparison with RNA-seq (Table 2). Therefore, these results suggest that miR167j and miR172c precursor lncRNAs could be responsive to drought stress.

**Table 2:** The lncRNAs were identified as miRNA precursors.

Gene_ID	maize miRNA precursor	% coverage	% identity	Up or Down On RAN-Seq
TCONS_00012662	MIR167j	100	100	Up
TCONS_00046477	MIR169d	100	100	Down
TCONS_00042984	MIR169h	100	100	Up
GRMZM2G420571_T01	MIR172c	100	100	Up
TCONS_00044116	MIR399b	100	100	Down
TCONS_00024310	MIR399e	100	100	Down
TCONS_00024309	MIR399e	100	100	Down
TCONS_00054544	MIR827	100	100	Down

### Characterization of ncRNAs for shRNA and siRNA

To identify drought-responsive ncRNAs as precursors of shRNAs or siRNAs, small RNA libraries of shRNAs and siRNAs were respectively mapped to the remaining 656 lncRNAs using Bowtie without mismatches. The results show that 62 lncRNAs are homologous to numerous shRNA reads and may hence be classified as shRNA precursors (Table S4). About 341 lncRNAs were homologous to numerous siRNA reads and hence classified as siRNA precursors (Table S5). In addition, we found that all of 62 shRNA precursors are homologous to siRNA library reads and may hence be classified as siRNA precursors. The ncRNAs are homologous to multiple types of small RNAs, which suggests overlap between sRNA biogenesis pathways at numerous loci. In plants, evidence of overlapping epigenetic regulatory pathways exists [37], [38].

**Figure 3:**
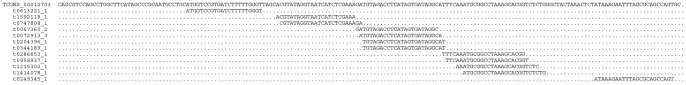
The TCONS_00012703 were mapped with multiple siRNA reads.

**Figure 4:**
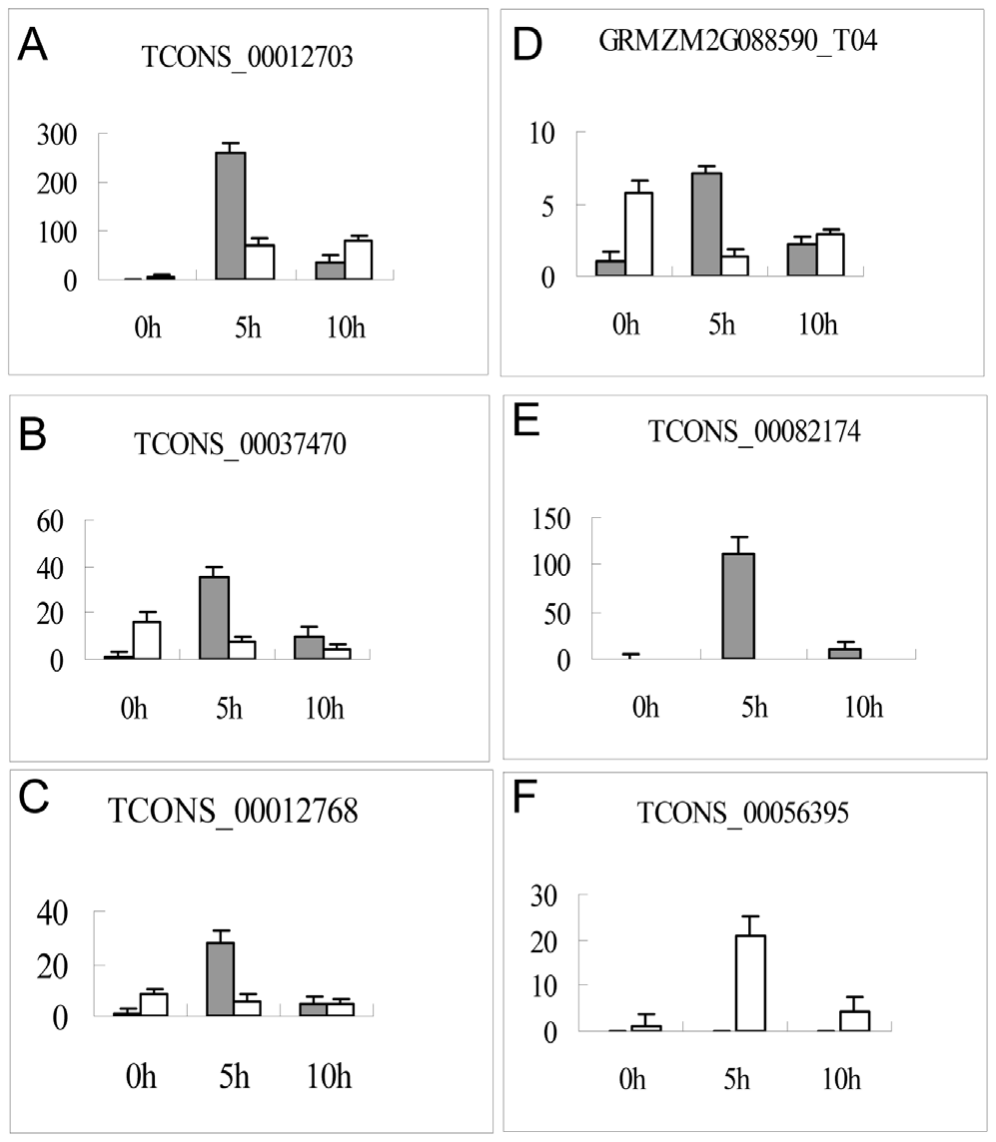
Quantitative analysis of 6 siRNA precursor lncRNAs in the leaves and roots using RT-qPCR at 0, 5 and 10 h. The gray bar denotes leaf samples and the white bar denotes root samples. GAPDH RNA was used as the internal control. Error bars indicate SD obtained from three biological repeats.

Based on the established mechanisms of biogenesis, siRNAs are expected to derive from a longer molecule cleaved into multiple sRNAs through specific endonuclease activity. To determine if multiple sRNAs are derived from siRNA precursor ncRNAs, 341 siRNA precursor ncRNAs were mapped to siRNA reads and subjected to further analysis. Results show that most of the siRNA precursor lncRNAs produced more than one sRNA (Table S5). For instance, Figure 3 shows that TCONS_00012703 is homologous to multiple siRNA reads. In addition, the RT-qPCR result of TCONS_00012703 RNA demonstrates that the expression level of the lncRNAs is relatively low in both non-stressed leaves and roots but accumulates to high levels after drought stress in both plant tissues (Figure 4A).

To validate these drought-responsive lncRNAs, qRT-PCR was performed to detect the expression profiles of 5 siRNA precursor lncRNAs randomly selected from the 279 lncRNAs at 3 time points of 0 h, 5 h and 10 h after drought treatment. Similar to the RNA-seq data (Table S2), the RT-qPCR results show that 4 siRNA precursor lncRNAs are upregulated in stressed leaves (Figure 4 B-E) except for a root-specific lncRNA of TCONS_00056395 (Figure 4F), and exhibit maximum expression in leaves stressed for 5 h. In addition, TCONS_00082174, which was only expressed in leaves, was not detected in roots, which suggests leaf-specific expression (Figure 4E). GRMZM2G088590_T04 which was downregulated in root stressed for 5 h, was upregulated in roots stressed for 10 h (Figure 4D). The remaining 2 lncRNAs, TCONS_00037470 and TCONS_00012768, were downregulated in drought-stressed roots (Figure 4B and C). These findings confirm that these siRNA precursor lncRNAs are responsive to drought stress in maize leaves and roots. Moreover, based on the differential expression profiles observed between leaves and roots, these lncRNAs may participate in gene expression regulation in different pathways within leaves and roots.

### Characterization of the remaining 315 lncRNAs

Among the 664 drought-responsive lncRNAs, the 315 lncRNAs that were not classified as small RNA precursor lncRNAs, including miRNAs, shRNAs and siRNAs precursors, were finally classified as other lncRNAs that are likely to function as longer molecules (Data S3). Among them, 167 were intergenic lncRNAs, 132 were intragenic lncRNAs, 10 were antisence transcripts and the remaining 6 were overlapped with parts of intragenic and intergenic regions (Table 3). To determine whether or not these lncRNAs could regulate the expression of protein coding genes as long molecules, excepted for 10 antisense lncRNAs, the remaining 305 lncRNAs were tested for homology to CDS sequences using BLAST with Plus/Minus patterns with >90% identity and >90% coverage. The results demonstrate that 7 lncRNAs could pair with 17 CDS sequences with near-perfect matches (Table 4), which suggests that these 7 lncRNAs might regulate the expression of 17 proteins by inducing transcriptional or post-transcriptional gene silencing.

**Table 3:** Classification of 664 drought-responsive lncRNAs.

	intergenic	intragenic	antisense	overlapping	Total
miRNA	6	1	0	1	8
shRNA/siRNA	52	5	4	1	62
siRNA	196	67	9	7	279
Other lncRNAs	167	132	10	6	315
Total	421	205	23	15	664

**Table 4:** The 7 lncRNAs were mapped to the 17 CDS sequences with near perfect match.

Query_name	Hit_name	Query_len	Sbject_len	Hit_len	Percent_identity	Percent_query_aligned	Percent_Sbject_aligned
GRMZM2G541687_T02	GRMZM5G828887_T01	1258	246	254	93.3	20.19	103.25
GRMZM2G541687_T02	GRMZM5G817336_T01	1258	183	181	96.1	14.39	98.91
TCONS_00012840	GRMZM2G367206_T05	357	2013	336	95.8	94.12	16.69
TCONS_00012840	GRMZM2G367206_T04	357	1182	336	95.8	94.12	28.43
TCONS_00012840	GRMZM2G367206_T03	357	1935	336	95.8	94.12	17.36
TCONS_00012840	GRMZM2G070807_T05	357	1866	336	90.5	94.12	18.01
TCONS_00012840	GRMZM2G070807_T03	357	1866	336	90.5	94.12	18.01
TCONS_00012840	GRMZM2G070807_T02	357	1536	336	90.5	94.12	21.88
TCONS_00012840	GRMZM2G070807_T01	357	1947	336	90.5	94.12	17.26
TCONS_00012924	GRMZM2G359904_T01	591	4095	591	95.4	100	14.43
GRMZM2G141152_T02	GRMZM5G850027_T01	684	345	345	100	50.44	100.00
GRMZM2G141152_T02	GRMZM2G364145_T01	684	291	269	92.6	39.33	92.44
GRMZM2G397297_T02	GRMZM2G509619_T01	1105	294	294	100	26.61	100.00
GRMZM5G805389_T01	GRMZM2G065989_T02	543	327	327	100	60.22	100.00
TCONS_00066999	GRMZM2G117281_T03	789	222	210	92.3	26.62	94.59
TCONS_00066999	GRMZM2G117281_T02	789	222	210	92.3	26.62	94.59
TCONS_00066999	GRMZM2G117281_T01	789	222	210	92.3	26.62	94.59

**Figure 5:**
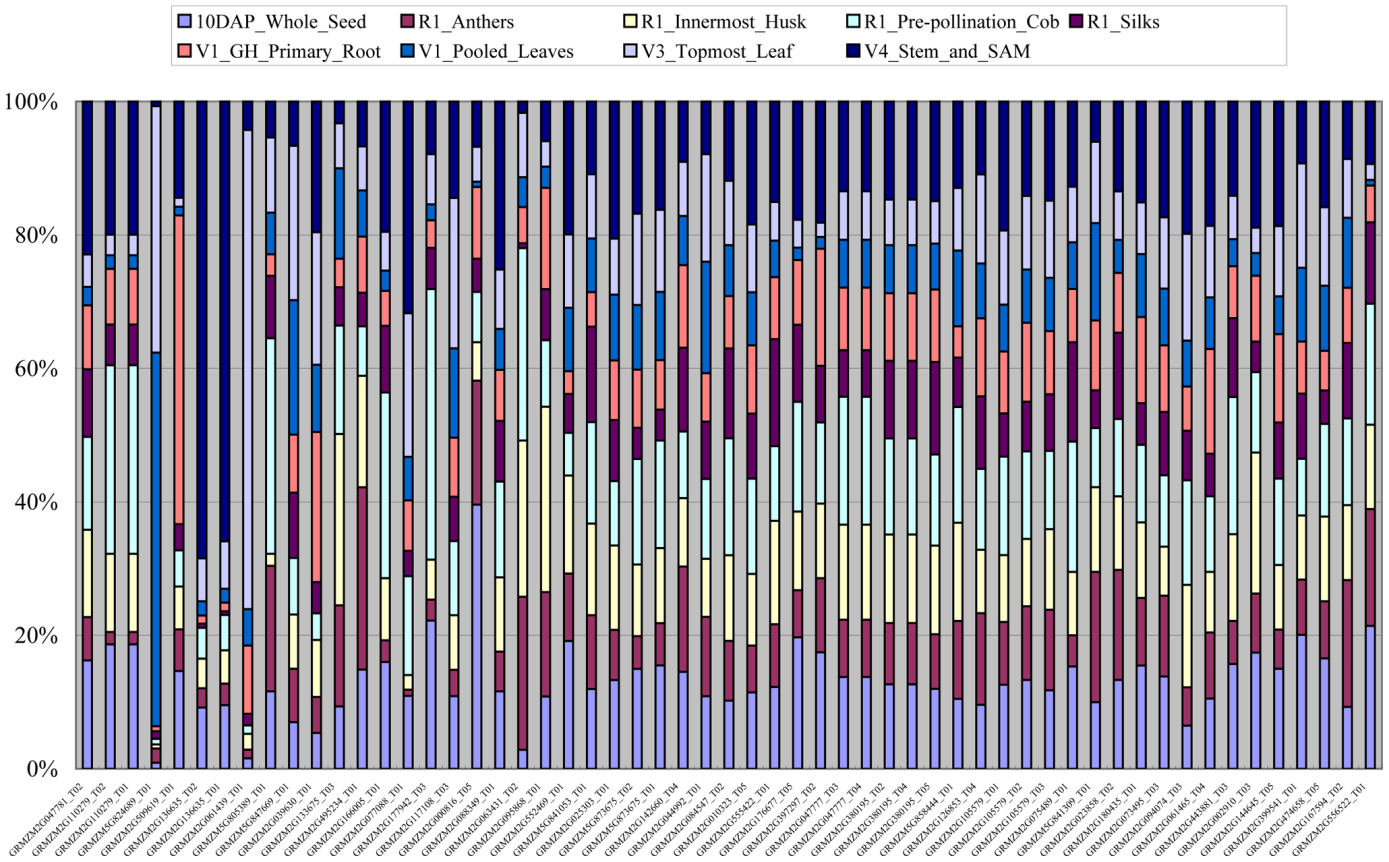
The tissue specific expression of 57 lncRNAs.

lncRNAs have been found to exhibit a wide range of functions. The transcription of certain lncRNAs is highly tissue- and temporal-specific, and their expressions may be responsive to certain stimuli. To analyze the tissue-specific expression of these 315 lncRNAs, the expression data of 70061 maize transcripts in 9 tissues (10DAP_Whole_Seed, R1_Anthers, R1_Innermost_Husk, R1_Pre-pollination_Cob, R1_Silks, V1_GH_Primary_Root, V1_Pooled_Leaves, V3_Topmost_Leaf and V4_Stem_and_SAM) were downloaded from the maizeGDB website, http://maizegdb.org/, and reversed to the raw expression values. The raw expression values of the 9 tissues were extracted based on GeneID. Ultimately, only 57 of the 315 drought-responsive lncRNAs could be obtained from the tissue expression data. According to the expression profiles of these 57 lncRNAs in 9 tissues, most lncRNAs exhibited tissue-specific expression (Figure 5, Table S6).

**Figure 6:**
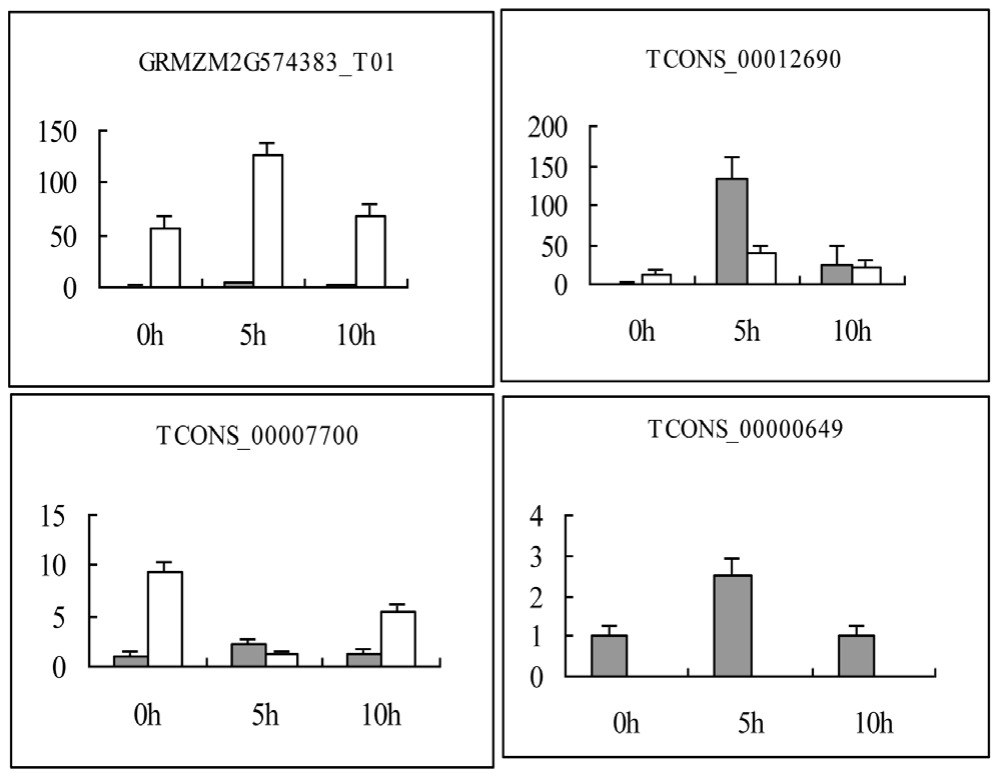
Quantitative analysis of 4 other lncRNAs in the leaves and roots using RT-qPCR at 0, 5 and 10 h. The gray bar denotes the leaf samples and the white bar denotes root samples. GAPDH RNA was used as the internal control. Error bars indicate SD obtained from three biological repeats.

Four lncRNAs, GRMZM2G574383_T01, TCONS_00012690, TCONS_00007700 and TCONS_00000649 were randomly selected from the 315 lncRNAs for expression analysis in maize leaves and roots at 3 time points of 0, 5, and 10 h after drought treatment using qRT-PCR. Similar to the RNA-seq data (Table S2), RT-qPCR results showed that the 4 lncRNAs were obviously upregulated in stressed leaves (Figure 6). TCONS_00000649 lncRNA exhibited leaf-specific expression, whereas the expression of the GRMZM2G574383_T01 was relatively low in leaves but very high in roots, suggesting a root-specific lncRNA. Furthermore, TCONS_00007700, which was upregulated in stressed leaves, was downregulated in stressed roots (Figure 6).

## Discussion

LncRNAs are a type of novel molecule with important functions in a wide range of biological processes, including developmental regulations and stress responses; nevertheless the detailed mechanisms involved in this biological processes remain largely unknown [39]. In this study, to identify the putative maize lncRNAs responsive to drought stress, a total of 664 out of 8449 differentially expressed transcripts in maize were identified as drought-responsive lncRNAs, including 126 known and 538 novel lncRNAs. In plants, small RNAs are an important class of noncoding RNAs for the regulation of gene expression at the transcriptional or posttranscriptional level. These sRNAs can originate from longer precursors that are processed by endonucleases, such as those identified by Dicer [40]. In this study, 8 out of the 664 drought-responsive lncRNAs were identified as precursors of miR167j, miR169d, miR169h, miR172c, miR399b, miR399e and miR827. RT-qPCR also confirmed that miR167j and miR172c precursor lncRNAs participate in maize drought-stress responses. These miRNAs have been reported as drought-responsive miRNAs in rice or maize in previous studies [41], [42].

Among the 664 drought-responsive lncRNAs determined, 315 lncRNAs including 10 antisenses and 305 non-antisenses were ultimately identified as other lncRNAs that are likely to function as longer molecules. To understand their function, the 305 non-antisense lncRNAs were tested for homology with CDS sequences. Results show that 7 lncRNAs could pair with 17 CDS sequences, which suggests that these 7 lncRNAs regulate the expressions of 17 proteins by inducing transcriptional or post-transcriptional gene silencing. Antisense transcripts can mediate gene silencing via transcriptional or post-transcriptional mechanisms, with the latter involving mRNA degradation. Antisense-mediated mRNA degradation likely generates siRNAs, and these siRNAs were not detected in the available datasets. The lncRNA candidates may produce siRNAs, but these siRNAs are not represented in the datasets used to create the databases for this study.

Numerous lncRNAs have been reported to participate in responses to a wide variety of stresses, including biotic or abiotic stresses. In *Arabidopsis*, the expression of 1832 lncRNAs has been changed after 2 h and/or 10 h of drought, cold, high-salt, and/or abscisic acid (ABA) treatments [43]. In wheat, 125 lncRNAs were also identified as powdery mildew-responsive lncRNAs and heat stress-responsive lncRNAs [44]. In this study, some lncRNAs were selected through their expression in both drought-stressed and control samples using RT-qPCT. Results demonstrate that all of the selected lncRNAs show apparent changes in their expression profiles in leaves stressed for 5 h or 10 h. In addition, several lncRNAs exhibited leaf-specific or root-specific expression. These results prove a previous finding that lncRNAs respond to abiotic or biotic stress via tissue-specific expression [25], [26].

## Supporting Information

Data S1 **The sequences of 1724 potential non-coding RNA identified by CPC.**
(RAR)

Data S2 **The sequences of identified 664 lncRNAs.**
(RAR)

Data S3 **The sequences of 315 lncRNAs that were not classified as small RNA precursor lncRNAs.**
(RAR)

Table S1 **Primers used in this study.**
(DOC)

Table S2 **The expression of 664 lncRNA in control and drought stress leaves.**
(XLS)

Table S3 **The lncRNAs were homologous to known lncRNAs in maize.**
(XLS)

Table S4 **The lncRNAs had homology with shRNA reads.**
(RAR)

Table S5 **The lncRNAs had homolgy with siRNA reads.**
(RAR)

Table S6 **The tissue specific expression of 57 lncRNAs.**
(XLS)
