# Supplementary material for: Identification of Maize Long Non-Coding RNAs Responsive to Drought Stress
Source: PLoS One. 2014 Jun 3;9(6):e98958. doi: 10.1371/journal.pone.0098958 (PMC4044008; doi:10.1371/journal.pone.0098958)
Supplement: Table S1 — Primers used in this study. (DOC) [file pone.0098958.s004.doc]

Table_S1 Primers used in this study.

| lncRNAs | Primer | sequence |
| --- | --- | --- |
| TCONS_00012662 | Forward primer | CTCTGGTCGAGATAGTAGCGTG |
|  | Reverse primer | TGCTGCCACGAGAAAATGA |
| GRMZM2G420571_T01 | Forward primer | ACAGTGGGTGCTTGCAGGT |
|  | Reverse primer | ATGAGTGAGTGTCTGCTGATGC |
| TCONS_00044116 | Forward primer | CATCAGCAGCAGGCAGTCA |
|  | Reverse primer | CAGGGCAAGGCCAGAAAT |
| TCONS_00024310  TCONS_00024309 | Forward primer | TCAGGAAATGGAATGCCTAAGC |
|  | Reverse primer | CACCATAGGCCGGTACAACA |
| TCONS_00054544 | Forward primer | AGCCACCATTCCATTCTCG |
|  | Reverse primer | CATGCACTGATGCGCCAC |
| TCONS_00012703 | Forward primer | TGCCTGCATGGTCCGTGA |
|  | Reverse primer | TGGGAAAAGCATAGGGTGAT |
| TCONS_00012768 | Forward primer | AGGGCAGCGATCAAGTCTC |
|  | Reverse primer | CCGCCTAAAAGGGGTGGT |
| TCONS_00037470 | Forward primer | ACAGATACACCACCGTCCAGC |
|  | Reverse primer | CCTCGGGCAGACTAATACCAG |
| GRMZM2G088590_T04 | Forward primer | CAGCGAATCCATCCCCATC |
|  | Reverse primer | CGAAGCCATCGGCGAAGA |
| TCONS_00056395 | Forward primer | GTCTCAGTGTAGTGCCTCCGTC |
|  | Reverse primer | CCACTCCCATTGCGTGTTG |
| TCONS_00082174 | Forward primer | AAGGATTGGATGCGTATGTAGG |
|  | Reverse primer | GAGCACAGATACACCACCGTC |
| GRMZM2G574383_T01 | Forward primer | CCAAACAGATGCCATGACAGA |
|  | Reverse primer | TTGCCTTCAGTGATGACCAGT |
| TCONS_00012690 | Forward primer | AAGCCCCACGGGAAAACA |
|  | Reverse primer | CCGCTGTTACCACCACCG |
| TCONS_00007700 | Forward primer | CAAGCCAGGTTCTTTCCCG |
|  | Reverse primer | GGTTGCTGCCATCCATGTTAC |
| TCONS_00000649 | Forward primer | GGGATGTCCATTTCAAGCC |
|  | Reverse primer | GCAGTCACAACGATGAATCAGA |
| GADPH | Forward primer | CCCTTCATCACCACGGACTAC |
|  | Reverse primer | AACCTTCTTGGCACCACCCT |
